# “He’s shouting so loud but nobody’s hearing him”: A multi-informant study of autistic pupils’ experiences of school non-attendance and exclusion

**DOI:** 10.1177/23969415231207816

**Published:** 2023-10-18

**Authors:** Laura Gray, Vivian Hill, Elizabeth Pellicano

**Affiliations:** Educational Psychology Service, Barnet, UK; 4901 UCL Institute of Education, 4919 University College London, London, UK; UCL Institute of Education, 4919 University College London, London, UK; Department of Clinical, Educational and Health Psychology, 4919 University College London, London, UK

**Keywords:** Inclusion, integration, school avoidance, school refusal, anxiety

## Abstract

**Background and aims:**

Children and young people on the autism spectrum frequently report a range of negative educational experiences and face disproportionally high rates of school non-attendance, including school avoidance and permanent exclusion, which can have a significant impact on their well-being as well as educational and broader life outcomes. To date, few studies have examined the full range of proximal (child, parent/family, school levels) and distal (community and society levels) barriers to ensuring the school attendance and the inclusion of autistic pupils. The current study sought to do just that by examining autistic young peoples’ school non-attendance and exclusion experiences from the perspectives of multiple informants.

**Methods:**

We recruited 12 autistic pupils, who had previously experienced school avoidance and/or exclusion, from one local authority in England, United Kingdom. We conducted semi-structured interviews with the young people themselves, ten of their parents, eight of their current teachers and nine local authority professionals, including six educational psychologists and three specialist autism teachers. We analyzed interviewees’ responses using reflexive thematic analysis.

**Results:**

Interviewees gave overwhelmingly negative accounts of autistic pupils’ school non-attendance and exclusion experiences. Our analysis identified a range of school-related factors they felt led to, or exacerbated, negative experiences in their former mainstream schools, and which ultimately led to their or their children's school non-attendance. It also went further to identify distal factors, including fragmented educational experiences, parents “fighting” against a complex bureaucratic system to secure appropriate education for their children, and limited professional involvement.

**Conclusions:**

Our findings emphasize the importance of examining the broader context in which autistic pupils are embedded and demonstrate that such pupils are able to successfully attend—and even enjoy—school when they receive the appropriate care and support.

**Implications:**

Schools and local authority professionals should seek to work in partnership with parents and autistic pupils to secure the necessary support for their inclusion in mainstream education. Government policy should support the provision of sufficient local authority professionals to adopt a more proactive approach to mitigate autistic pupils’ avoidance of and exclusion from school.

School is central to children's lives. A significant minority of children, however, miss out on school. In England alone, in 2017–2018, 11.2% of pupils showed persistent school absence (missing >10% of school sessions) (Department for Education (DfE), 2019), and these numbers have continued to grow almost every year since (DfE, 2022a). Some reasons for missing school are “non-problematic”, including medical appointments, short-term illness or cultural observances; others, however, are “problematic”, occurring due to school “refusal” (hereafter referred to as “avoidance”), parental withdrawal and fixed-term (suspension) or permanent exclusion (expulsion). Persistent non-attendance can have a significant, negative impact on children's mental health (Epstein et al., 2019; McAra & McVie, 2010), academic achievement (Hancock et al., 2013; Rosenbaum, 2020) and long-term employment prospects (Madia et al., 2022). It can also cause distress for families (Want, 2020) and place extra demands on school staff (DfE, 2022b; Ofsted, 2022).

There are some pupils who experience problematic school non-attendance at disproportionately high rates (DfE, 2019, 2022a) —including autistic^1^ pupils. There is, however, exceedingly little known about these young people's experiences. The current study adopted a multi-informant approach to address this, specifically focusing on a group of autistic young people whose persistent absence led to their eventual non-attendance or exclusion from school.

## Autistic experiences of school

Autistic children and young people frequently report negative experiences of school (Goodall, 2020; Heyworth et al., 2021; Humphrey & Lewis, 2008; Makin et al., 2017; Williams et al., 2019). They regularly encounter sensory challenges within the school environment (Jones et al., 2020); complex social expectations and interactions (Williams et al., 2019); social isolation—despite often reporting a desire to fit in (Carrington & Graham, 2001; Humphrey & Lewis, 2008)—and bullying (Aubé et al., 2020; Maïano et al., 2016); a plethora of transitions (Makin et al., 2017; see also Nuske et al., 2019); and limited attention to their specific needs, strengths and preferences (Heyworth et al., 2021; Makin et al., 2017; Wood, 2021). These challenges are further compounded by the prevailing view that autistic children are “difficult” to include (House of Commons Education and Skills Committee, 2006), including from mainstream teachers, who report lacking confidence in understanding their autistic students (Ravet, 2018; Roberts & Webster, 2020; Robertson et al., 2003).

In this context, it is perhaps unsurprising that government data from England (DfE, 2019) and Wales (John et al., 2022) show that autistic pupils have elevated rates of absence and persistent school absence, placing them at further risk of academic underachievement (Keen et al., 2016) and mental health complications (Crane et al., 2019). Recent work has attempted to delineate further the nature of autistic pupils’ non-school attendance. Totsika et al. (2020) sampled 486 parents from the United Kingdom, who reported their autistic children missed five days of school of a possible 23 days (22%) in March 2017. School avoidance accounted for almost half (43%) of these absences, while 9% was due to school exclusion. Similarly, in an Australian sample of 106 parents of autistic pupils, Adams (2022) found children missed six full days of school across a four-week period (30%), with 9.4% missing at least one full day due to school exclusion, including being sent home either for their behavior or because the school could not support their needs.

In the case of school avoidance, non-attendance happens in the knowledge of the parent and where the parent has made reasonable attempts to ensure the child's attendance. It is often underpinned by mental health issues, including internalizing problems and/or emotional distress (Heyne et al., 2019)—and has been re-conceptualized as “emotionally-based school avoidance” (Halligan & Cryer, 2022). In the case of school exclusion, pupils can either be formally excluded for a short period (fixed-term exclusion or suspension) or excluded from school altogether (permanent exclusion or expulsion) because of their behavior^2^. If pupils have been permanently excluded or cannot be accommodated by another school through a “managed move”, they will be provided with an alternative provision (DfE, 2016; see also Bagley & Hallam, 2015). Exclusion and managed moves are known to place strain on pupils’ mental health and can be linked to feelings of shame, stigmatization and rejection (Harris et al., 2006; Parsons et al., 2011).

Melvin et al. (2019) recently proposed the Kids and Teens at School (KiTeS) framework to understand the factors that influence school absenteeism. They drew on Bronfenbrenner's (2005) bioecological systems model, which emphasizes that a person's development is influenced by multiple, interacting contexts or “systems”. At the most proximal or immediate level, the KiTeS Framework identified a range of child (e.g., age, gender, mental/physical health, disabilities), parent (e.g., parental stress, mental/physical health, parenting style), family (e.g., family functioning/dynamics) and school factors (e.g., school climate, home-school relationships) linked to school attendance or academic engagement. Melvin et al. (2019) also highlighted a series of more distal factors at the community (e.g., community support, transport, school type/structure) and societal levels (e.g., government policy, cultural values, neighborhood characteristics) thought to shape absenteeism, especially in potentially vulnerable populations.

The limited studies thus far examining autistic pupils’ school non-attendance and exclusion experiences have shown they are indeed influenced by a range of individual-, family-, and school-related factors, in line with this Framework. School exclusion has been found to be more likely for autistic pupils from sole-parent, unemployed and well-educated homes (Totsika et al., 2020; though see Adams, 2022), while school avoidance has been linked to child anxiety (Adams, 2022; Munkhaugen et al., 2019), increased child age (Adams, 2022; Totsika et al., 2020) and experiences of bullying (Bitsika et al., 2021; McClemont et al., 2021; Ochi et al., 2020). Three qualitative studies have further identified school-related factors contributing to autistic pupils’ school exclusion experiences from the perspective of the pupil themselves (Brede et al., 2017; Sproston et al., 2017), their parents (Brede et al., 2017; Martin-Denham, 2022; Sproston et al., 2017) and teachers in their post-exclusion alternative provision (Brede et al., 2017). All three studies highlighted the challenges of learning within noisy and complex mainstream environments, teachers’ often-limited understanding of autistic pupils’ needs and preferences, negative peer relationships, inappropriate and ineffective responses to pupils’ behavior (including physical restraints), and poor parent–school relationships. Informants in these studies also emphasized the psychological distress experienced both by the pupil and their family owing to the events leading up to the exclusion and the exclusion process itself.

## The current study

The current study sought to understand the school non-attendance and exclusion experiences of autistic pupils by extending existing qualitative work in two important ways. First, through individual semi-structured interviews, we examined autistic young peoples’ experiences from their own perspectives—alongside those of their parents, their teachers and local authority professionals. Adopting this multi-informant approach ensured the possibility of identifying both proximal (child, parent/family, and school levels) *and* distal factors (community and society levels) influencing the school non-attendance/exclusion of autistic pupils, the latter of which have hitherto remained unaddressed in previous studies. Second, we adopted a broad definition of school non-attendance and exclusion—including permanent exclusion, managed moves, school avoidance and parents removing their child from school—to ensure it captured the full range of experiences of autistic pupils who had persistent, problematic attendance and experience of leaving a mainstream setting due to unmet needs.

## Method

### Participants

To recruit autistic pupils who had previously experienced school avoidance or exclusion, we identified all eight alternative provisions—which provide education to pupils with a diverse range of needs, who are unable to attend mainstream schools—within one local authority in the East of England, inviting their autistic pupils to take part. Twelve autistic pupils (10 boys, 2 girls), aged between 13 and 16 years, from two alternative provisions agreed (Table 1).

**Table 1.** Pupil participant details.

Pupil	Age (yrs)	Gender	Ethnic background	Parent-reported diagnoses	Parent participant	Type of exclusion
PU1	15	Boy	White British	ADHD^a^	Mother (PA1)	Managed move
PU2	16	Boy	White British	ASD^b^	Father (PA2)	Managed move
PU3	16	Boy	White British	Asperger syndrome	Mother (PA5)	Permanent exclusion
PU4	13	Boy	Mixed	Autism, tics, ADHD	Adoptive mother (PA3)	Permanent exclusion
PU5	17	Boy	White British	ASD/APD^c^	Mother (PA4)	Self-excluded
PU6	15	Boy	White Irish	ASD	Mother (PA6)	Managed move
PU7	14	Girl	Mixed	Autism	—	Self-excluded
PU8	14	Boy	White British	ASD/ADHD/dyspraxia	Mother (PA7)	Removed by parent
PU9	17	Boy	White British	HFA^d^, motor tics, anxiety	Mother (PA9)	Self-excluded
PU10	15	Boy	White British	ASD/DAD^e^	Foster mother (PA8)	Permanent exclusion
PU11	15	Girl	White British	Autism	—	Managed move
PU12	16	Boy	White Irish	Autism, dyspraxia, dyslexia	Mother (PA10)	Self-excluded

Notes: ^a^ADHD: Attention Deficit/Hyperactivity Disorder.

^b^ ASD: Autism spectrum disorder.

^c^ APD: Auditory processing disorder.

^d^ HFA: High functioning autism.

^e^ DAD: Disorganized attachment disorder.

All participants had received an independent clinical diagnosis of an autism spectrum condition, except for one pupil. Although this pupil had not obtained a formal diagnosis, we included him in the study given that his parents and school staff felt he demonstrated clinically significant autistic features. All children, including this latter pupil, obtained scores on the Social Communication Questionnaire (Rutter et al., 2003; see below) either at or above the threshold for autism (cut-off score of 15) (M  =  22.78, SD  =  4.89, range  =  15–29). Pupils’ scores on the Wechsler Abbreviated Scales of Intelligence—Second edition (Wechsler, 2011) ranged from very low (score of 45) to superior (score of 142) (M  =  96.75, SD  =  27.84), indicating wide variation in intellectual functioning.

Of the 12 pupils, 10 of their parents also took part, including seven biological mothers, one biological father, one foster mother, and one adoptive mother. We also recruited eight teachers (four male, four female), who knew the pupils well, as well as nine local authority professionals, including six educational psychologists (six female) and three specialist autism teachers (three female). These professionals did not know personally the participating pupils but spoke to the broader context.

### Procedure

Semi-structured interviews were carried out individually with each participant by the first author between June 2017 and February 2018. We asked each participant a series of open-ended questions about school exclusion, including the factors that led to it, the exclusion process itself and its impact (see Supplementary Materials). The questions were reworded to suit their different roles. For example, we asked pupils and their parents questions about their experiences of mainstream, of school exclusion and of their reintegration into their current provision; we asked teachers to focus on the pupil in the study and how they were supported within the current, alternative provision; and we asked educational psychologists and specialist autism teachers about their experiences supporting autistic pupils, especially those at risk of non-attendance.

We also adopted two inclusive approaches to supplement autistic pupils’ semi-structured interviews (see Supplementary Materials). First, following the Life Grid Method (Jalali & Morgan, 2017), pupils mapped their educational experiences by writing key words next to time points on a prepopulated timeline. Second, following the Drawing the Ideal Self technique (Kelly, 1955; Moran, 2001), we used the Drawing the Ideal School activity in which pupils were asked to draw two pictures—one of their non-ideal and another of their ideal schools—naming elements of the classroom and describing the other pupils and teachers. These activities were designed to help establish rapport, assist in the recall of key events, reduce the social demands of the interview, and facilitate discussion (Bell, 2005; Chase, 2005; O’Connor et al., 2011).

#### General procedure

Ethical approval was obtained from the Human Research Ethics Committee at UCL Institute of Education. All participants in the study provided written informed consent, including pupils. Child consent was viewed as a “continuous process” throughout the study (Lloyd et al., 2006).

All pupil interviews were conducted individually in their alternative provision in a quiet room, with a familiar staff member nearby. The WASI-II (Wechsler, 2011) was always completed first, followed by the in-depth interview. Parent interviews were conducted in a place most convenient to them, including in their child's current provision (n  =  2), their home (n  =  4), over the phone (n  =  3) and in a public place (n  =  1). Parents completed the SCQ (Rutter et al., 2003) ahead of, or during, the interview. Teacher interviews were conducted in their workplace (n  =  6) or over the phone (n  =  2). Interviews with educational psychologists and specialist autism teachers were conducted individually and face-to-face at their workplace.

### Data analysis

All thirty-nine interviews were recorded and transcribed verbatim, except for the interview for one pupil, who declined to be recorded. In this case, the researcher relied on extensive note-taking during their interview. We followed Braun and Clarke's (2019) approach to reflexive thematic analysis within an essentialist framework, in which our goal was to report the meanings and experienced reality of participants. Our analysis was informed by our experience and training in educational psychology. We used an inductive, bottom-up approach to identify patterned meanings within the dataset. Following transcription, the first author immersed herself in the data, taking reflexive notes on striking and recurring observations and applying codes to each transcript. Data were initially coded separately by informant (young people, parents, teachers, professionals), although it soon became apparent through discussion that the codes and potential themes were common across informants. All transcripts were therefore combined and re-coded where necessary. All three authors met multiple times to discuss initial codes, consider researcher preconceptions, resolve discrepancies and decide on the final themes and subthemes. Analysis was therefore iterative and reflexive (Braun & Clarke, 2019).

## Results

**Figure 1.**
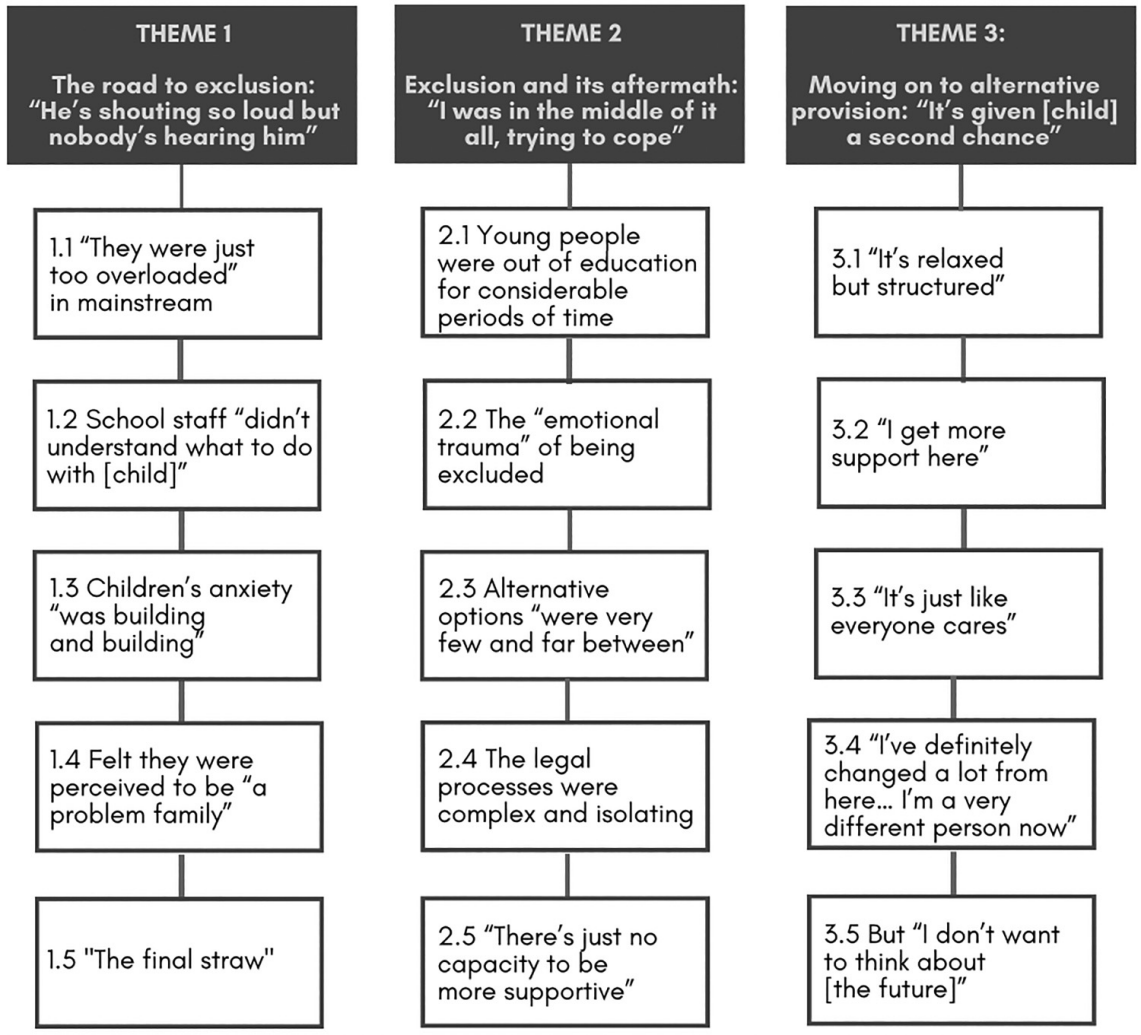
Participants’ experiences of school non-attendance and exclusion: themes and subthemes.

Overall, pupils reported overwhelmingly negative accounts of their previous mainstream schools, which appeared to result largely from challenges posed by their school or broader context. We identified three themes and associated subthemes (Figure 1), which are also numbered below and presented in bold and italics, respectively. Illustrative quotes are also provided, attributed via participant IDs (PU: autistic pupil; PA: parent; T: teacher; AT: specialist autism teacher; EP: educational psychologist).

### THEME 1. The road to exclusion: “He's shouting so loud but nobody's hearing him”

Parents and teachers often described their autistic children and students as “academic” [PA10], “very bright” [PA4], “phenomenally intelligent” [T3] and “very articulate” [T8], but nevertheless felt that “*they were just too overloaded”* in mainstream (subtheme 1.1)—in multiple ways. Young people spoke of the sensory overload, how the classrooms were “very big” [PU10], “the lights were too bright” [PU1], which made it difficult to “filter out background noise and deal with distractions… so I basically just mentally logged out” [PU5]. They also repeatedly described the social overload, how the sheer “number of people was just intimidating” [PU6]—in part because they didn’t “get on with a lot of the people” [PU11] and were “bullied” [PU10], and in part because “I didn’t know what they were going on about” [PU7]. These “social struggles” [T5] were exacerbated by the desire to fit in, to be “just like one of them [peers]” [PU4]. They also felt overloaded academically, especially with homework, which “was extremely stressful”, and which often led “to lots of sanctions” leading to “lots of distress” [EP4]. Many young people attributed their “hate” for homework to “a straight separation between school and home” [PU6]: “In Year 7, I tried to do homework, but I was having like breakdowns and crying every other day; it was not a happy time” [PU5].

Adult interviewees overwhelming agreed that “all that pressure” [PA2] of the “academic and social and emotional demands” [EP1] combined with “the amount of stimulation, the noise, the movement and the different changes going on” [EP2] are “what led to him falling down” [PA10]. This sense of overwhelm was often expressed as “unwanted behavior” [PA1], especially during unstructured times, “when [child] doesn’t know what to do with himself” [PA1] and “when he would get into little scraps” [PA2]. Young people agreed: “less freedom makes it easier—cos there's not enough time to do anything” [PU1].

This was compounded further by reports that school staff “*didn’t understand what to do with [child]” (subtheme 1.2)*. Young people described how they would have benefited from more “support and help” [PU2], “a bit more one-to-one” [PU1], and “stuff made more structured instead of it being relaxed” [PU3]. Instead, they commonly reported that staff were “unknowing of either my condition or needs” [PU6] and, as a result, felt unfairly treated: “like, they were just always telling me off, they’d always assume I was up to something” [PU3], or who were “arbitrarily strict on some people, which made me just feel stressed and I just refused to engage” [PU5]. One young person conveyed her experience:

The teachers shouted a lot, and they were like really aggressive… they would start shouting at me because I tend to zone out a lot as well, and I always ask the person next to me what's going on, so then I got told off for that, and I got sent out a lot. [PU7]

Adults lamented that “the teachers were lacking understanding of [child's] condition and therefore didn’t know how to get the best out of him” [PA4] and that school staff can “have very rigid views” [AT2], “which sounds funny because obviously that what's we talk about with our [autistic] children” [AT3]. They agreed that schools often responded to “children who present in a challenging way” [AT3] by focusing “on the negative things about [child]” [PA2] and being “so heavy-handed” [PA7]. They spoke of how their children were physically restrained: “when he couldn’t do time out, they’d hold him down on the floor” [PA3]. They were also reportedly told by school staff, including headteachers, that they “didn’t feel [child] was autistic, he was just badly behaved”. Consequently, children regularly experienced unofficial—even illegal—exclusions, including being told “he’d be better off at home today’” [PA9]) and excluded from school events (e.g., Christmas carols service, sports days) because “he's too naughty” [PA7].

Support for autistic students reportedly varied enormously between schools: “some have so much in place for autism and are fantastic and some aren’t, [despite] all basically having the same intake, same sort of kids” [AT2]. While some professionals felt a “real willingness to try and accommodate the needs of children with autism” [EP6], most felt that schools did not always “carry out the advice we suggest” [AT2] or implement statutory guidance (“quite clearly the teachers never even read his statement” [PA9]), often misunderstood “what's driving the child's behavior” [AT3] and failed to “apply their knowledge very well” [EP4]. They wanted schools to have a “more empathetic view of the child” [EP3], to provide “the security of somebody who is a critical friend, who is constant, fair and that he trusts” [PA7], “so that they feel safe in that environment” [AT2]. One young person pleaded for greater understanding: “people think autism is just a learning difficulty, but autism is much more than that” [PU8].

Anxiety was described as “a common thread” [AT2] for autistic children's negative experiences of school. One teacher portrayed his student's anxiety as “very tangible, almost like you felt you could reach out and touch it” [T3]. Interviewees described how their own or their *children's anxiety “was building and building” (subtheme 1.3)*; “it was getting worse and worse throughout Year 8” [PU8]. Parents described how they “didn’t realize he was in such a state of anxiety” [PA4] in part because their children “couldn’t articulate his emotions and he didn’t understand them himself” [PA4] and in part because “for me, anxiety and stress don’t really manifest typically” [PU5]. Indeed, some interviewees described their children's anxiety “manifested in aggression” [PA6], “he just seemed to be more agitated, more angry, oppositional” [PA9], which, for one family, culminated “in incidents where I’ve been told to call an ambulance and the police” [PA6]. Others spoke about how their or their children's anxiety manifested in physical ways, including “nausea and vomiting” [PA4], “stool withholding, chronically,” [PA7], problems sleeping, including lying “awake at night because of stress and anxiety” [PU5], and “panic attacks, and shaking uncontrollably… he was in a constant fight or flight mode” [PA9]. One young person described:

Everything sort of has a trickle-down effect for me, basically if one thing tips me over the edge, it will start to overflow a little bit, it will start breaking, and then it just sort of shatters and everything goes wrong. [PU6]

Prolonged experiences of anxiety eventually resulted in their children “being desperately unhappy” [PA5] and eventually “shutting down… it became too much for him and stayed in his bed all day everyday” [PA6]: “I had to take him to casualty I was so worried about him” [PA4]. They reported self-harm and suicide attempts, as well as “mental health breakdown… you just couldn’t reach him, he was catatonic, non-communicative” [PA10]. Professionals felt these crises were often preventable, particularly “understanding that if they’re behaving in a certain way, it's not necessarily that they can help it; it's that their anxiety is getting in the way” [EP5]. Parents agreed: “if his anxiety is managed, the violence and aggression and all those things are completely nullified” [PA6].

Autistic children's elevated anxiety and its varied manifestations often meant that parents received phone calls “pretty much every day” [PA1] and “relentless” emails about their child's “disruptive behavior” [PA3] or “his well-being’” [PA9]. One parent explained how she was constantly on alert: “you know it's never gonna be good when you see the school number; something's going to have happened and it's out of your control” [PA7]. Although some parents reported schools being “very supportive” [PA4], this was often only after much parent advocacy. Most spoke of how they were “constantly battling” [PA7] with schools to get their children the support they needed and, as a results, felt they were *perceived to be “a problem family” (subtheme 1.4)* [PA6].

Professionals repeatedly spoke of the importance of building positive relationships with schools so that “parents feel supported and listened to” [AT2]. But parents felt this happened all too rarely because “you’re just a parent” [PA9]. Instead, most reported increasingly adversarial interactions with schools: “the correspondence throughout the time he was at secondary school just got nastier and nastier from the school” [PA7]. Professionals explained schools’ reactions: “when people accumulate negative experiences about that child… everybody gets slightly fed up with it, they feel worn out by it” [EP6]. These negative perceptions could also be transferred to parents: “I’ve seen placements breakdown because that [parent-school] relationship has broken down… to the point where the school can no longer tolerate the interactions with the parent” [AT3].

For all our young people and parents, these issues eventually came to a head where the young person was either formally excluded from school, or “decided to leave… it's effectively the same thing” [AT1]. Professionals explained that “*the final straw” (subtheme 1.5)* often happened at the beginning of secondary school, when “they can end up with 16 different teachers with different expectations—that transition is actually huge” [AT2]. They explained further that the nature of the exclusion often depends on whether the student shows “externalizing or internalizing behaviors… it's the ones that have the angry outbursts that tend to get permanently excluded or managed moved. And it's those who have anxiety and really struggle that then refuse to go back to school” [EP3].

For our young people who fell into the former group, they were excluded for “setting off fire alarms, stuff like that” [PU2], getting “involved with the police because they were saying I punched someone in the face, and I didn’t” [PU8], being “caught vaping—but just general naughtiness, they were already on the edge there, just waiting for any excuse to kick me out” [PU3] and “hitting a boy on the head with the tennis racket—he was 4 at the time” [PA7]. In these cases, young people and families were acutely aware that the school “had had enough of him and they wanted him out” [PA2]. One professional admitted that “very often staff morale is quite low and, if they’re getting pressure, it's easier to get rid of the problem than try and solve it” [EP3]. Another suggested they lacked the resources to support children: “I’ve seen more pupils this year permanently excluded or at risk of permanent exclusion, managed move than I’ve ever seen… schools are just saying ‘we’ve not got the funding to put in the extra support you’re all talking about’” [EP4].

For the latter group who instead decided to leave school, it was often because young people had severe mental health difficulties, which reached the point at which there was “pure unwillingness to go, I just couldn’t go in there” [PU6], were “on the verge of suicide, because I felt like that's not where I am supposed to be” [PU8], “started to get more depressed and stuff” [PU7], “chose not to attend because he was vomiting all the time… he wanted to go to be normal, to have the same tasks as everyone else but he just couldn’t cope with it” [PA4], or “had slid into a deep depression and wouldn’t even get out of bed” [PA10]. Professionals sympathized with parents’ decisions to remove them from school: “if your child was so anxious and being so traumatized by going to school, would you put them through that? It's difficult” [EP1].

### THEME 2. Exclusion and its aftermath: “I was in the middle of it all, just trying to cope”

Being excluded—either through informal, fixed-term or permanent exclusions or through school avoidance—meant that young people were *out of education often for considerable periods of time (subtheme 2.1)*. Some young people reported that, prior to exclusion, school “put me on a reduced timetable” [PU7] or “a different timetable to normal” [PU3]: “I was in school but only for about two hours” [PU4]. Others reported their non-attendance “in Year 9, sort of just started plummeting, because of the homework thing… more and more often I would just not go in or feel extremely sick” [PU5]. Another young person recounted being in hospital and “out of school most of Year 9 and 10 because I started to get bad, because I have mental health issues” [PU7]. Parents also reported their children being “signed off [school] through anxiety… since he started secondary school, he didn’t have much schooling” [PA9]. These avoidance and informal exclusion experiences meant that young people were “actually out of education for a long time” [PA5], usually between 1 to 3 years, oftentimes not accessing any education at all: “he's missed a good two years of education” [PA8]. One young person explained:

I didn’t want to be out of school… but I was off cos my headteacher was trying to find out what to do with me, my mum was at home just doing this, and they had loads of meetings. Meanwhile, I was in the middle of it all, just trying to cope. [PU4]

Understandably, parents and professionals were extremely concerned about *the “emotional trauma” of being excluded (subtheme 2.2)*: “it's been a nightmare” [PA1]. Some described being “removed from a very challenging situation where their needs weren’t being met” [EP3] as “a relief actually” [PA1]. Nevertheless, they were worried about “lots of gaps in his knowledge” [PA5] and the “significant impacts on their mental health and well-being” [AT3]. Adult interviewees were concerned in particular about how “it affects children's self-confidence” [EP5], as “it builds into this view that people are scary, schools are bad” [EP3], as well as the damage done to “their sense of belonging and their self-esteem, if they are always being told that they’re doing things wrong” [EP1]”. They sympathized with how “completely rejected… they must feel” [AT1]. While some professionals described how things could turn around “if they’ve been moved to the right setting, and they can maybe put it down to the first school not having been the right place for them” [EP5], most felt that “there's nothing good that comes from young people being out of school” [EP4], and that “the more they are isolated, the harder it gets to go back” [EP3].

The distress of being excluded extended to families: “it has been really, really difficult for us as a family” [PA1]. Parents reported having “to give up work, because it was impossible for me to maintain a job and do the Education, Health and Care (EHC) Plan^3^ application at the same time” [PA4], and how the exclusion process had negative effects on their own mental and physical health: “I cried, I had shingles every single year because I was so stressed out about it” [PA3]. Some parents reported also a sense of guilt either “for trying to force him to go to school when he clearly was utterly terrified” [PA4] or because “you’re responsible for this naughty child, you’re obviously not parenting them properly” [PA7]. This parent further described how the family also became: “very quickly excluded from relationships with other parents, and so it's a very socially isolating experience.”

One key reason why the process was drawn out was because *alternative options “were very few and far between” (subtheme 2.3)*. Parents were “constantly told that there wasn’t a provision for [child]; there isn’t anything we can do with him because while he has learning difficulties due to his autism, he is actually not disabled in that [academic] way” [PA6]. Consequently, they “wasted time” [PA9], “emailing lots of schools, looking at schools all over the place” [PA6], “just filling in ‘til they [school] found him somewhere” [PA3]. Some families wanted inclusive, mainstream settings “but it was very, very difficult to find anywhere. A lot of schools will pay lip service to inclusion—what they say and what they do are very different things” [PA7]. Her child agreed: “I applied to a lot of mainstream schools, most of them didn’t want me to go there… they thought I had a lot of issues” [PU7]. Other families wanted more specialist provision, with the possibility of “smaller classes and sometimes to have one-to-one time” [PA1]. Professionals were again sympathetic to parents’ predicament: “there is nowhere for those children to go—it's mainstream or special, that's it, there's no middle ground” [AT3]. While some professionals felt that specific provision for autistic young people without intellectual disability “is lacking” [EP2] in the local authority, others felt it was the school's responsibility to “provide accessible education for all children” [PA6]: “I suppose I have got in the habit of saying to schools, ‘well, you just need to adapt your practice’” [EP4].

The impact on families was further compounded and prolonged because *the legal processes were complex and isolating (subtheme 2.4)*. Parents felt that “[child] was entitled to go to his local state school, he was entitled to have his needs met… but you get big push back” [PA10]. Some young people were not in receipt of an EHCP prior to their exclusion, sometimes because “the panel couldn’t make a decision because they couldn’t understand why somebody so academically able was not able to attend school” [PA4]. In the words of one parent, “that's when the battle began” [PA7]. Parents reported that “going through the EHC process and getting all the documentation and so on was five months of torture—the panel kept bouncing backwards and forwards” [PA4]. Some families “got turned down for an alternative placement… it was a very long fight” [PA9]. Another family “took the local authority to court, for failure to provide an education—they got their act together, put an EHCP in place” [PA6]. They described the process as isolating—“you do feel like you’re on your own” [PA3]: “there's no help given whatsoever, and that is incredibly difficult” [PA4].

Professionals agreed with their sentiments. While they felt their role is “about working with the family and empowering the families” [EP1] and “being proactive rather than reactive” [AT3], in reality, they were often asked to support the process “a bit late” [AT1], “because settings don’t always flag up until there's a real problem” [AT3]. One professional recounted that she “had been called in to see one young man who's been school refusing for seventeen months—and he's never come my way” [EP3]. Another advisory teacher reported that “some of the EHCPs we get… the number of times I’ve read that paperwork and thought, ‘oh my God, why have we never even been called in?’” [AT2].

Despite professionals’ concern about not being able “to intervene earlier, before it reaches crisis point” [EP1], they were adamant that “*there's just no capacity to be more supportive” (subtheme 2.5)*. They described how they often do not have time “to really form proper relationships with the children” [AT1], “do individual work” [EP4] or “more observations” [EP6]. Educational psychologists reported they “are meant to offer a consultation service” [EP3], as well as “linking, facilitating between parents, the young person and school and getting everyone to talk to each other—a kind of conduit” [EP5]. There was resounding agreement, however, that while they would like to “be more strategic” [AT2] and “do more intensive work, we’re all just so stressed—we have so many children on caseload” [AT1]. Instead, they reported “getting bogged down with things like EHCP applications” [AT2], “statutory work and tribunals” [EP3]. One professional summed it up: “it's all about capacity” [EP6].

### THEME 3. Moving on to alternative provision: “It's given [child] a second chance”

At the time of interview, most young people had been accessing education in the alternative provision for at least one year, while others had only recently transitioned. In sharp contrast to their previous school experiences, parents and young people were overwhelmingly positive about their placement: “it's all pretty good at the moment” [PU3]. One key factor in its apparent success was that “*it's relaxed but structured” (subtheme 3.1)*. The “smaller school space” [PU3] with “less people” [PU1] meant that there “were less distractions, and less opportunity for [student] to make wrong choices” [T2] and staff “can actually cater more for what the child's needs” [PA9]. Young people spoke repeatedly of how “everyone was very relaxed” [PU10], and the staff are “friendly” [PU4]. This student explained: “say, if you can’t understand something, they’re like, ‘oh, do you wanna maybe do this another day and go onto a different task?’”. Parents agreed that their children's new placements were more flexible: “they don’t mind too much if he has a day off… it's like, ‘oh, that's a wobble, move on’” [PA6]. Another parent also appreciated how the teachers “are very good at not saying, ‘right, these are the rules’; they gradually over time get the kids to modify their behavior” [PA5]. This flexibility made young people feel that they were “able to learn” [PU5]. Only one pupil felt that mainstream school “challenged me [him] a lot more” [PU1] and his parent agreed: “I think they could probably stretch him more academically” [PA1].

Perhaps as a result of having “had a pretty rough ride” [T4] in their previous schools, young people appreciated how “*I get more support here” (subtheme 3.2)*. They felt “the nature of the teaching is one of the biggest benefits… it's one-to-one and if there are any things that aren’t exactly clear, you can ask immediately rather than just having to give up” [PU5]. Other young people agreed that if a student needs help, “the teacher will be able to focus all of their attention on them for a bit” [PU4].

Parents went further to suggest that it was more than simply one-to-one support; it is also the ways in which staff “tailor [child's] learning to suit his particular abilities” [PA4]: “they still follow a curriculum but do it in a way that engages the child rather than this is the set way we’ve got to do it. It's about knowing a child's needs” [PA9]. Teachers explained that “we try and create timetables around the students’ preferences—they’ve got to do Maths and English as a non-negotiable, then as much as we can we do in discussion with them” [T3]. Young people experienced this personalized approach as greater “freedom in the classroom” [PU9].

Young people also felt their teachers understood their differences: “they’ve obviously got the understanding of autism and all the different things as well, you know—it's not just autism, there's ADHD, there's lots of different things” [PU6]. Teachers explained that “before, I thought [autism] was something I had to learn to manage to help… but now I see it's about making sure my lessons are relevant” [T7]; “it's just knowing to tap into what they’re good at and then helping them to flourish” [T3]. They were careful not to “profess to actually know anything about [autism] because unfortunately you get the impression that nobody really knows everything about it” [T6]. Instead, they “we’re very open to new ideas… we’re always sort of changing to meet the needs of the kids we’ve got” [T7]. They reported being “open to learning about autism in different ways, including from autistic voices… but I think I could be better, I’m always looking to be much better than I am” [T3]. Teachers also described “constantly reviewing and revising what we do for each young person” [T5] using a collaborative approach: “every single day after school, we run through every child we have—and we will talk through any positives or negatives for the day, and on the back of that, we’ll develop strategies” [T6]. In so doing, “you sort of always feel supported—if there's something I’m struggling with, I can always go to a colleague, and they’ll give me some ideas” [T7].

Critically, young people emphasized the importance of strong, trusting relationships with their teachers at their alternative provision: “*it's just like everyone cares” (subtheme 3.3)*. They spoke of how they valued “caring and fun teachers” [PU6], who “I can get along with” [PU5] and “don’t shout—at my old school, all of my other teachers used to shout a lot because they didn’t get it” [PU7]. Young people also highlighted how “some teachers go out of their way to help” [PU1], “help sort out problems” [PU10], and how they in turn felt valued: “they were just like willing to listen to what I had to say… I felt you could trust everyone here” [PU2]. Parents overwhelming agreed that “that trust element” [PU9] was crucial for their children's success. Parents described how their children have “started to open up a lot more… because they’ve taken the time to get to know him” [PU1], to “build up this connection” [PU9]. One parent admitted that her son's tutor “probably knows more about how [child] is feeling than I do!” [PA4]. They described further how their autistic children “are quite alert of how people perceive them… having teachers that want to be there and will give everything to help that child, I think that's the key” [PA9]. As a result, young people felt “safe and looked after” [PU3] in their new environments: “I like the fact it makes you feel at home” [PU4]. This level of care had a positive effect on parents, too, as it meant they were “relaxed and not worried at all” [PA3]: “to know that you can send your child somewhere where you’re not going to spend the day worrying, because you trust them to get it right… because I know they care” [PA7].

Teachers described their students in positive terms (“he's a really lovely kid” [T8]) and emphasized how the young people enjoy “working with teachers that he can develop a relationship with and who understand him and his needs” [T3]. This level of care also extended to parents. Teachers described “having a really strong relationship” [T7] with parents who they “contact [parents] on a weekly basis preferably by telephone… the idea is that we have quite a trusting open relationship” [T2]. One teacher elaborated: “[parents] are a great resource—it sounds terrible to talk about them like that, but it seems bloody stupid dunnit? If you’re trying to get to know someone to ignore the person whose been with them the longest” [T6]. Professionals, too, noted that “it's so much about the relationship” [EP6].

It is not surprising, then, that many of the young people reportedly thrived in their alternative placements. As one young person [PU6] put it, “*I’ve definitely changed a lot from here… I’m a very different person now” (subtheme 3.4)*. Teachers felt that “he's quite a different lad from the one who came here in September” [T1], that they were “in a better place” [T4] and “generally very happy to be in school” [T8]. Parents “could see a change in [child]” [PA2] in multiple ways. Young people were “better at dealing with anxiety” [PU5] and “much more open to understanding his emotions” [PA4], which had positive effects on their behavior, including fewer “big meltdowns” [PA3] and being “not so aggressive—[school] have managed to bring out the child I knew was there” [PA6]. These positive changes extended to their academic work, in which many had “just flourished” [T6]. Parents were surprised—and proud—that their children, who had “missed a big chunk of his education” [PA8], were now studying for GSCEs and A-levels^4^: “the fact that he's learning at this level is a major thing” [PA3]. They also reported their children were “more independent” [PA10] and were “becoming more social” [P4], which “is lovely because he never really had friends before” [PA3]. Young people felt this too: “I feel like I mean something to my friends, like I’m just like one of them” [PU4].

These changes were partly attributed to how “settled” [T4] and “trusting” [T8] the young people had become, and how the staff had “made him feel good about what he could do” [PA2] and had given students strategies “to cope better with the stressors” [T3], helping “them make changes” [T6]. Parents felt that previously their children “didn’t know how to cope with being autistic” [PA8], but the staff at school have “enabled him to be positive and be who he wants to be, be how he feels he is inside” [PA6]. One teacher expanded: “when we picked him up, he was 15 going 16, but it was almost like that kid had had no time to actually explore his own self and capabilities” [T6]. As a result, they felt “he's definitely more confident” [PA10], “feeling worthwhile” [PA6], and “more able to express his likes and dislikes and the things that he would like to experience” [T3]. Young people valued that they were able to make choices with their timetables and that they felt heard: “he likes the fact that… people are listening to him” [T5].

Overall, parents recounted how “I just don’t think anyone's really given him a chance in life” [PA8] but this placement “has given [child] a second chance and turned his life around” [PA10]: “it's the best thing that has ever happened to him” [PA5]. One parent summed it up: “it's astounding really—he has a future now whereas before there was no future for him” [PA6].

While young people's newfound confidence and trust in the people supporting them made our interviewees “realize, actually, he can do really well” [PA1], many were nevertheless anxious about “when he goes from here” [T1]. In the words of one young person: “*I don’t want to think about [the future]” (subtheme 3.5)*. Indeed, young people described themselves as “the kind of person that doesn’t think ahead” [PU4] or who tends “to avoid thinking about it because I have a very depressive outlook on my life” [PU7]. Their parents agreed: “I don’t think he has any concept of the future” [PA4]. Many parents wanted them to stay at the provision for as long as possible because “he's missed so much education and also being autistic, in certain areas of life he's very immature, and I just think to stay in education gives him a chance to catch up” [PA8]. Parents were worried that their children haven’t had “a chance to know what he wants to do [once he leaves school], cos he's so behind and most of his time's been spent struggling and coping with anxiety” [PA9]. They were worried about their vulnerability—both in terms of “going back to being how he was very quickly” [PA4] and of “people taking advantage of him” [T2].

Teachers understood why “parents want them to stay as long as possible, even when it's not necessarily in the best interest of the kid” [T8]. They discussed their many attempts to start that transition as early as possible “so that we’re not completely cutting the apron strings” [T4]. While some professionals cautioned that “the mainly one-to-one teaching is not ideal, as they’re missing out on all that social experience” [EP3], teachers were cognizant about “not wrapping autistic kids up into such a protective bubble” [T4], noting how “we’ve gotta keep them with us but then gradually sort of push them further away from us so that they actually go off and fly on their own” [T4]. They felt confident they were working towards “preparing them for life” [T6], “equipping him to deal with what he's going to face” [T8], trying “to build links with other secondary schools and national colleges as well” [T2]. Wherever young people ended up, parents and teacher were clear that they would probably need high levels of support: “whether that support comes from me, from social care, from a charity, from somebody taking him under their wing, or whatever; he would need a certain level of understanding with what he can and what he can’t do” [PA6]. While some were pessimistic (“to be fair, the outlook's very gloomy for a kid like him” [PA7]), others were more hopeful for the future: “he now thinks he has a future, which he didn’t before—he had no aspirations or anything… now, he's talking about college” [PA8]. Young people felt this, too: “I really don’t know how colleges work so I can’t really say how they could, like, adapt to me… but I’m gonna go to college” [PU1]. Teachers were also optimistic: “to recognize he's kind of on a journey so not to see the A-levels necessarily as the end of this journey, and then just really looking and trying to prepare him for that” [T3].

## Discussion

Autistic pupils and their parents often report adverse mainstream schooling experiences (see Horgan et al. (2023) for a review). Our participants’ accounts were no different. Autistic pupils and parents identified a range of school-related factors they felt led to, or exacerbated, negative experiences in their former mainstream schools, and which ultimately led to their non-attendance and/or exclusion. Many of these proximal factors, including sensory and social overwhelm, the perceived lack of understanding of autism by school staff, negative staff attitudes and problematic staff responses, as well as poor, often adversarial, home–school interactions, strongly echo previous work (Brede et al., 2017; Martin-Denham, 2022; Sproston et al., 2017). Our participants also emphasized additional school-based factors that have been less well articulated by existing research (see Sproston et al., 2017, for an exception), including their previous school's lack of flexibility with school rules (including homework), and the challenges with unstructured times, which can be socially demanding and anxiety-provoking for autistic pupils, and can also place them in precarious situations with their peers.

Our study also went further to identify distal factors that affected our participants’ school non-attendance (Melvin et al., 2019), including fragmented educational experiences, a complex bureaucratic system that parents struggled to navigate as they tried to secure appropriate education and support (via EHCPs) for their children, and limited professional involvement. The lengthy delays in accessing alternative provision and resultant—and often-significant—periods of time spent out of education (see also Brede et al., 2017; Sproston et al., 2017) represent serious issues for autistic pupils, who are already at risk of poor academic (Keen et al., 2016), mental health (Crane et al., 2019; Mutluer et al., 2022; Simonoff et al., 2008) and broader life outcomes (see Pellicano, Fatima et al., 2022, for review). The effects of being out of education for so long combined with the need to “fight” to ensure their children's rights were far-reaching, as parents described the debilitating effects on their own mental health and capacity to work (Brede et al., 2017; Martin-Denham, 2022).

Our professional participants also raised concerns about the level of government funding for supporting autistic pupils and others with special educational needs and disabilities (SEND) as one additional distal factor potentially contributing to autistic pupils’ negative school experiences. This has become a serious issue in England, with a recent House of Commons Education Select Committee (2019) declaring that “there is simply not enough money in the [SEND] system to provide for the scale of demand” and the pressures of inadequate resources are frequently emphasized by school staff on the ground. A survey of mainstream teachers in England found that their greatest concern was on limited resources to support inclusive education for children with SEND, especially the lack of availability of specialist and support staff, funding and appropriate infrastructure (Warnes et al., 2022). It is not difficult to see how such pressures might intersect with more proximal, school-related factors—and might go some way to explain our participants’ negative experiences in mainstream placements. For example, inadequate funding for supporting autistic pupils might pose constraints on school leaders and teachers’ capacity to adopt a flexible approach to inclusion. Such funding pressures might also affect staff morale, as well as staff attitudes towards inclusion, with one recent study demonstrating that teachers who believed they had inadequate resources to facilitate inclusion of their autistic pupils held more negative attitudes about their inclusion than teachers who did not hold such beliefs (Leonard & Smyth, 2022).

Such negative attitudes are, in turn, likely to influence the quality of home-school relationships and may lead to more adversarial interactions. Our participating parents reported a profound sense of isolation resulting, at least in part, from the lack of support from schools and local authority professionals—a sentiment with which our professional participants regretfully agreed. Local authority professionals can, in theory, play a key role in providing advice to schools about working effectively with autistic pupils and their families, facilitating positive relationships between home and school and, critically, advocating for parents and supporting schools to meet the needs of autistic pupils (e.g., Clarke et al., 2017). Our professionals were adamant that this proactive approach was necessary to prevent what they saw as the ever-increasing exclusion of autistic pupils. Yet, they also described that it was difficult to implement in practice. Sometimes this was owing to discouraging interactions with schools, who often alerted local-authority professionals to potential issues far too late. Mostly, however, it was attributed to a lack of capacity, due to broader constraints (a “mountain of statutory work”). A recent government report suggests these constraints are intensifying. Lyonette et al.'s (2019) survey of educational psychology services across England highlighted increasing pressures. Almost all local authorities surveyed reporting experiencing greater demand for educational psychology services than could currently be met. They also reported an increase in workloads with a concomitant decrease in the range of work, focusing predominantly on statutory work—a sentiment to which our professional participants attested.

Despite all of these challenges, what was perhaps most striking about the current findings—and occurred across school avoidance and exclusion—was autistic pupils’ *successful* re-integration into education. The reports of autistic pupils, their parents and their alternative-provision teachers clearly demonstrated that, under certain conditions, autistic pupils can engage in—and even enjoy—school. They repeatedly told us they benefited from smaller class sizes, which caused less social and sensory overwhelm than the larger class sizes of typical mainstream schools. The nature of the relationships with their alternative-provision teachers was particularly prominent. Autistic pupils reported feeling respected, listened to, and supported by their alternative-provisions teachers—a level of care they reported rarely experiencing in their previous mainstream schools (see also Brede et al., 2017; Goodall, 2018; Hummerstone & Parsons, 2021). Teachers, too, reported a fondness for their pupils and had high aspirations for them. They sought to provide tailored support within a structured environment that was sufficiently flexible to grant, and foster, autistic pupils’ autonomy. Autistic pupils themselves valued this flexibility and support, and the opportunity to have some control over their learning (see Heyworth et al., 2021). This emphasis on reciprocity and mutual understanding attained by our participating teachers and their pupils is extremely encouraging—and appears to overcome what Milton (2012) called the “double empathy problem,” the idea that there is a misalignment between the minds of autistic and non-autistic people, which can explain why they often struggle to understand one another. Importantly, these fundamentally *empathetic interactions* appeared to have positive implications on autistic pupils’ mental health and well-being and, often to their parents’ surprise, their academic progress.

The alternative-provision teachers’ care of their autistic pupils extended also to their parents, with whom they maintained regular contact and built trusting relationships. It is well known that successful and effective partnerships between parents and school staff can have a positive impact on autistic pupils’ experiences of school (Azad & Mandell, 2016; Azad et al., 2016; Garbacz et al., 2016; see Lilley (2019) for a review). Yet, parents of autistic children all-too-often report poor communication channels with schools, adversarial relationships with teachers and a lack of two-way partnership (Lilley, 2019; McNerney et al., 2015). Furthermore, despite feeling they know their children best, parents often describe feeling not listened to and excluded from classrooms, resulting in them feeling isolated and unsupported (Lilley, 2015; Makin et al., 2017). Our parent participants certainly described these feelings about their children's previous mainstream schools. But this was not the case with the alternative-provision teachers, whose sense of openness and willingness to learn appeared to foster positive partnerships between families and schools—just as it had done with the children themselves. The causes of this discrepancy are, of course, unclear, but may include the impact of training, professional development opportunities, school leadership, resourcing and the background motivations of teachers choosing different career pathways (see Cook & Ogden (2022) for discussion on some of these issues).

Some of the preconditions for successful school *in*clusion, especially maintaining low staff-to-student ratios, might be challenging to achieve in regular mainstream classrooms. But having teachers who are responsive to, and accepting of, their students’ individual strengths, interests and needs and develop caring relationships with them that are sustained across time should not be insurmountable. These qualities map directly onto a relational or ethics-of-care-based view of education (Noddings, 1984, 2010), which has long suggested that teachers develop caring relationships with their students and foster a caring capacity in them, too. Such a view of education, which emphasizes relationships and reciprocity and sees “interdependence as a necessary part of the human condition” (Gary & Berlinger, 2020, p. 56), has rarely been the focus of attention for those who are autistic (though see Fernandes (2019) for an exception)—probably due to long-held misperceptions that autistic people are not inclined to develop connections with others (Chevallier et al., 2012; though see Pellicano, Brett et al., 2022, for discussion). Our findings clearly suggest that strengthening care and connections in school, and being responsive to specific needs, has the potential to grant autistic pupils the respect and agency they require to lead flourishing lives (Pellicano, Fatima et al., 2022). Future research, especially with school leaders, should seek to determine whether these responsive attitudes and practices can be replicated in mainstream schools with autistic children at risk of non-attendance and/or exclusion.

## Limitations

This study is not without its limitations. First, this study focused only on two provisions in one particular local authority in England. Although caution is warranted regarding generalizing the findings beyond this specific context, especially in the context of our qualitative approach, it is nevertheless noteworthy that similarly negative exclusion experiences and the school-based factors contributing to them have been reported in different parts of England (Brede et al., 2017; Martin-Denham, 2022; Sproston et al., 2017). Second, although the proportion of autistic pupils (17%) from an ethnic minority mirrored the local context (19%), it was not, however, reflective of autistic pupils excluded from school across the country, which disproportionately occur for minority ethnic children (DfE, 2019). The already-harrowing experiences of exclusion reported by autistic pupils in the current study, therefore, may be an *underestimate* of those who experience multiple forms of marginalization. Third, while we captured multiple perspectives on autistic pupils’ exclusion experiences for this study, one notable omission was the perspectives of teachers and school staff from the excluding mainstream schools. Future studies should examine mainstream teachers and school leaders’ experiences of including (and excluding) autistic pupils to understand the constraints from their perspective, with particular focus on investigating in greater depth the potential interaction between the proximal and distal factors described above.

Finally, our study was not designed to compare the type of school exclusion autistic pupils experienced, namely school avoidance vs. permanent exclusion. It is noteworthy, however, that the school-level influencing factors, especially overwhelming school environments and limited trusting relationships with teachers and school staff, were remarkably similar across exclusion types, and participants often directly attributed their apparently disparate behaviors (withdrawal vs. externalizing behaviors) to the same issue, namely elevated levels of anxiety. There is a distinct lack of research on both types of school exclusion, especially school avoidance, which has likely increased substantially since the COVID-19 pandemic, particularly for socially-disadvantaged pupils (Centre for Social Justice, 2022). An important avenue for future research should be to identify which specific proactive supports might have the most effective impact on shaping autistic pupils’ school pathways.

## Conclusion

Autistic children and young people have a right to receive an education in their local mainstream school. Our findings emphasize the importance of examining the broader context in which autistic pupils are embedded (Pellicano & den Houting, 2022)—and that, though there are considerable proximal and distal barriers to ensuring the school attendance and inclusion of autistic pupils, these barriers are not insurmountable. Government policy should support the provision of sufficient local authority professionals to adopt a more proactive and preventative approaches, including implementing interventions or other measures to mitigate autistic pupils’ exclusions early in secondary school, before anxiety levels become extreme and attitudes and behaviors—on the part of all parties—are entrenched.

## Supplemental Material

sj-docx-1-dli-10.1177_23969415231207816 - Supplemental material for “He’s shouting so loud but nobody’s hearing him”: A multi-informant study of autistic pupils’ experiences of school non-attendance and exclusion Click here for additional data file. Supplemental material, sj-docx-1-dli-10.1177_23969415231207816 for “He’s shouting so loud but nobody’s hearing him”: A multi-informant study of autistic pupils’ experiences of school non-attendance and exclusion by Laura Gray, Vivian Hill and Elizabeth Pellicano in Autism & Developmental Language Impairments

## Notes

1. Throughout we use “identify-first” language (“autistic person”), rather than person-first language (“person with autism”), because it is the preferred term of many autistic people (see Kenny et al., 2016) and is less associated with stigma (Bottema-Beutel et al., 2021; Gernsbacher, 2017).

2. They might also experience informal exclusions, which can include exclusions from school trips/events, asking parents to collect their child from school or to keep them at home for reasons other than physical illness, including when their carer/teaching assistant is unavailable. These informal—and illegal—school exclusions seem to be alarmingly common (Ambitious about Autism, 2016; Atkinson, 2013; National Autistic Society, 2017) and are not officially recorded as exclusions.

3. An Education, Health and Care (EHC) Plan is a legal document that details the child's needs and education, health and social care support that the local authority in England has a duty to provide.

4. General Certificate of Secondary Education (GCSE) is a UK academic qualification that students take when they are 15 or 16 years old, at the end of Year 11. A-Levels (Advanced Level qualifications) are a UK subject-based qualification for students aged 16 and above.
