# Supplementary material for: “He’s shouting so loud but nobody’s hearing him”: A multi-informant study of autistic pupils’ experiences of school non-attendance and exclusion
Source: Autism Dev Lang Impair. 2023 Oct 18;8:23969415231207816. doi: 10.1177/23969415231207816 (PMC10583514; doi:10.1177/23969415231207816)
Supplement: sj-docx-1-dli-10.1177_23969415231207816 - Supplemental material for “He’s shouting so loud but nobody’s hearing him”: A multi-informant study of autistic pupils’ experiences of school non-attendance and exclusion [file sj-docx-1-dli-10.1177_23969415231207816.docx]

**Supplementary materials**


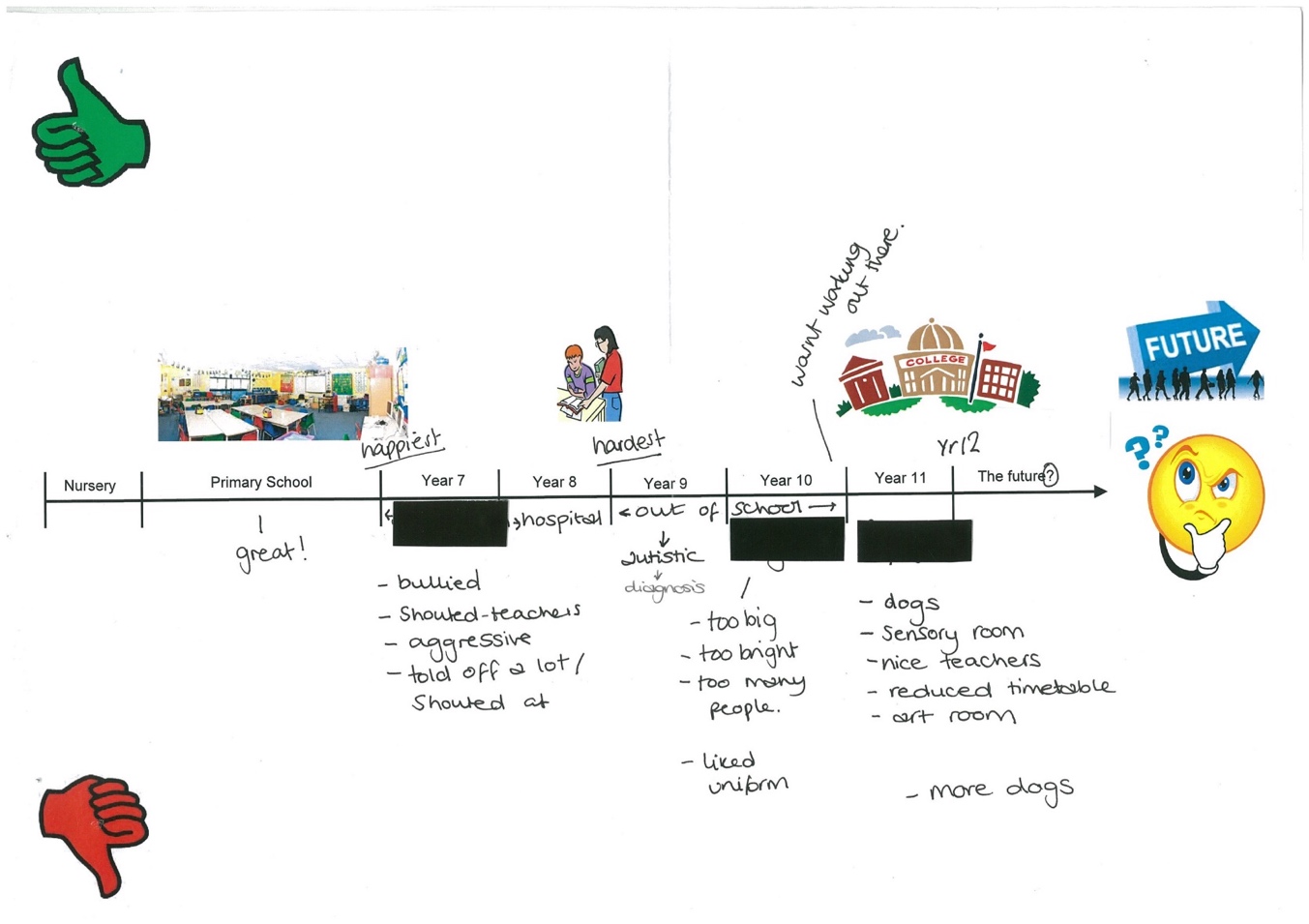


**Supplementary Figure 1.** Example of a completed Life Grid.


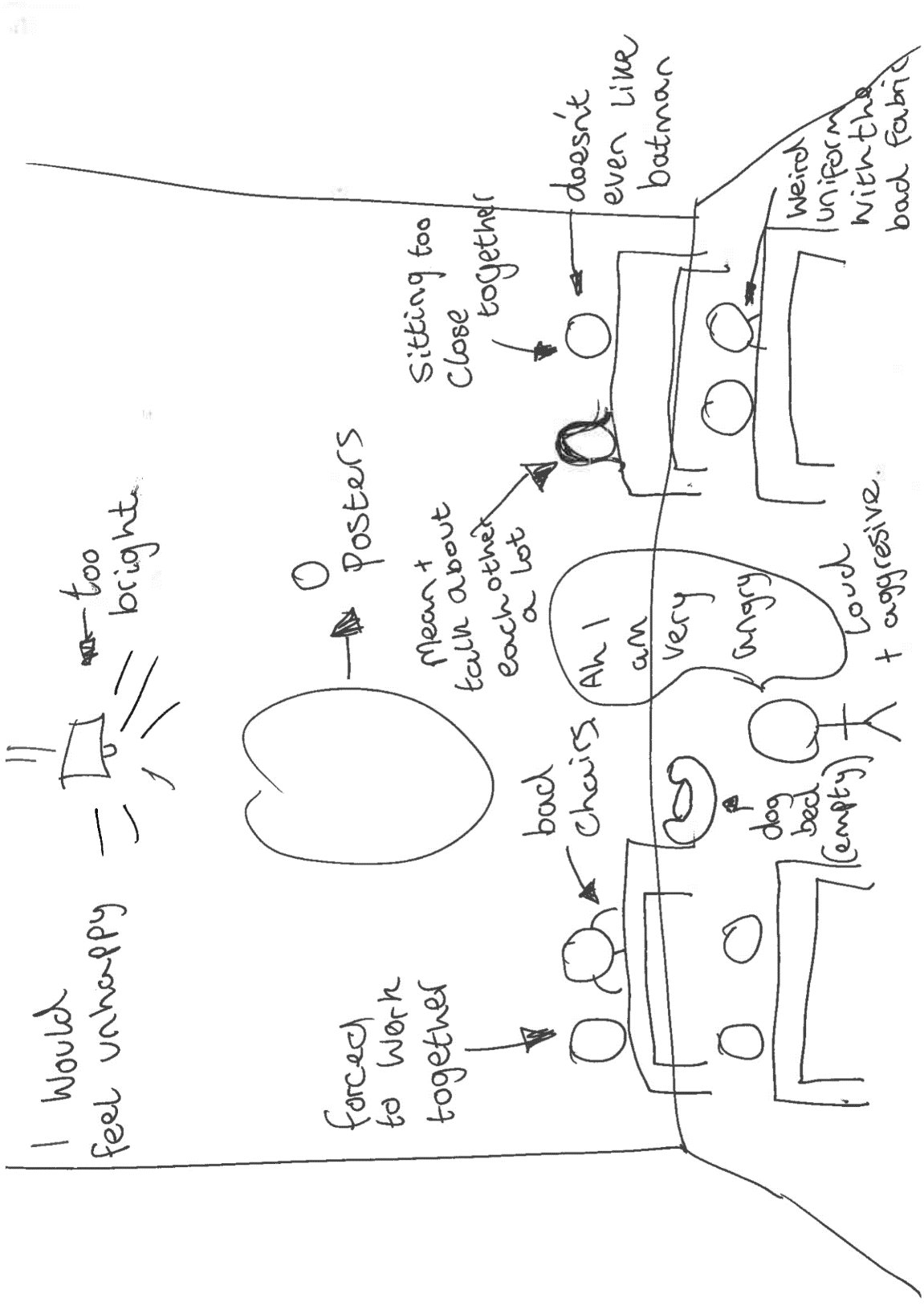


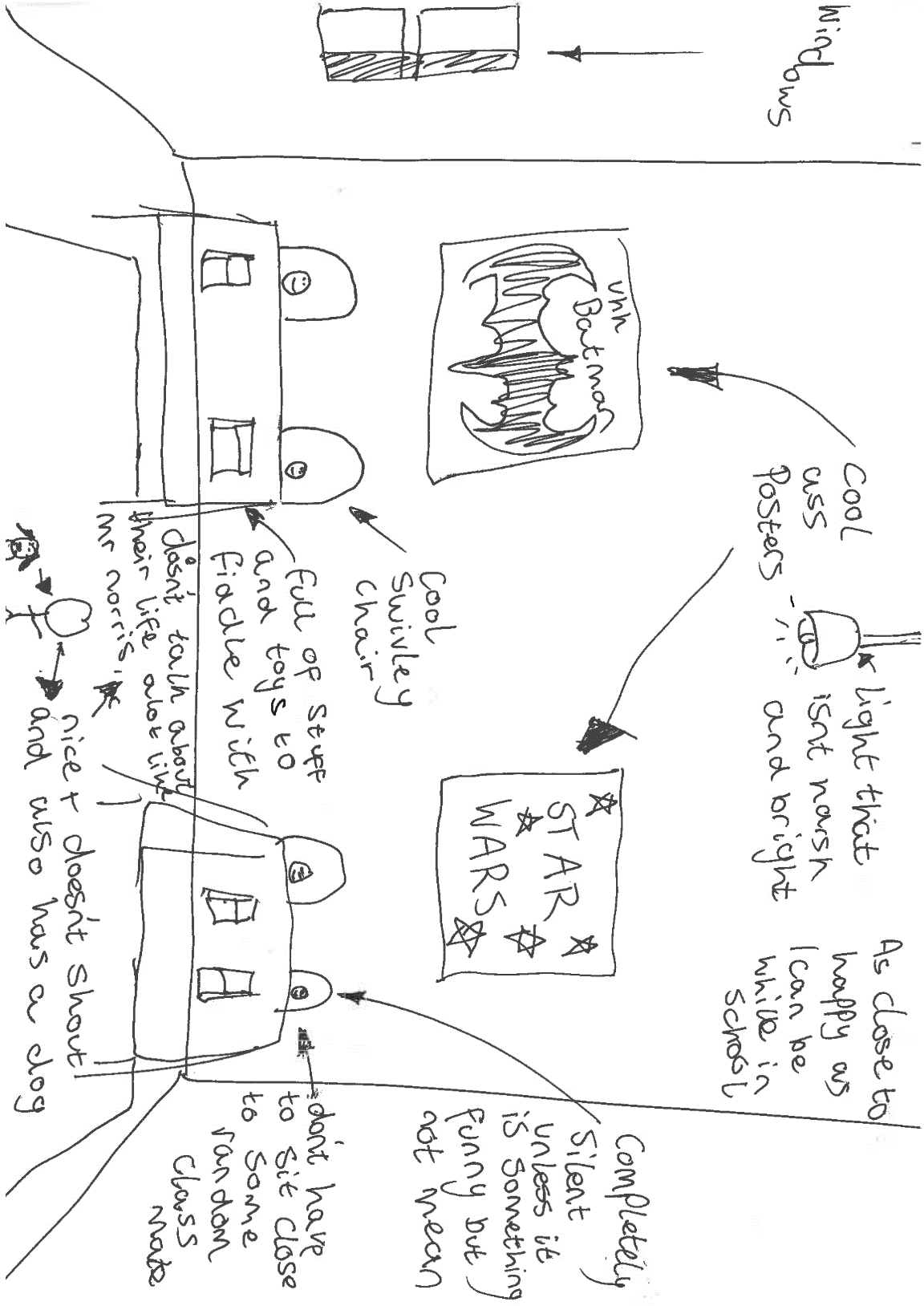


**Supplementary Figure 2.** Examples of the Ideal School Activity, including a non-ideal school (top) and ideal school (bottom) completed by one young participant.

**Supplementary Table 1.** Pupil semi-structured interview schedule with activities.

| **Life Grid**  This is called a life grid, it’s a way of helping you to talk about the different schools you have been to (show them life grid)  Current school  1. How long have you been coming to this school? (draw across these ages)  2. Tell me about (current school)  a. What do you like about (current school)?  b. What do you find difficult about (current school)?  c. What do the teachers do to help you here?  d. Is there anything else you would like your teachers to help you with?  Previous school  3. What school did you go to before you came to this school (draw on life grid)  4. How long did you go to that school for?  5. Tell me about (previous school)  a. What did you like about (previous school)?  b. What did you find difficult about (previous school)?  c. What did the teachers do to help you in (previous school)?  d. Is there anything else the school could have done to help you?  Now look at the timeline  6. When were you happiest in school?  7. When did you find school the hardest?  Hopes and Aspirations  8. How long will you be in this school?  9. Where do you hope to go next?  10.What are you hoping to do when you leave school?  a. What might help you get where you want?  b. What kind of support, if any, will you need to achieve your goals in life?  Is there anything missing from your life grid, for example other schools you have been to?  **Ideal School Activity**  Part 1: Drawing the kind of school you would not like.  The school. Think about the kind of school you would not like to go to. This is not a real school. Make a quick drawing of this school in the middle of this paper. Tell me three things about this school. What kind of school is this?  The classroom. Think about the sort of classroom you would not like to be in. Make a quick drawing of this classroom in the school. Draw some of the things in this classroom.  The students. Think about some of the students at the school you would not like to go to. Make a quick drawing of some of these students. What are the students doing? Tell me three things about these students?  The adults. Think about some of the adults at the school you would not like to go to. Make a quick drawing of some of these adults. What are the adults doing? Tell me three things about these adults.  Me. Think about the kind of school you would not like to go to. Make a quick drawing of what you would be doing at this school. Tell me three things about the way you feel at this school.  Part 2: Drawing the kind of school you would like.  The school. Think about the kind of school you would like to go to. This is not a real school. Make a quick drawing of this school in the middle of this paper. Tell me three things about this school. What kind of school is this?  The classroom. Think about the sort of classroom you would like to be in. Make a quick drawing of this classroom in the school. Draw some of the things in this classroom.  The students. Think about some of the students at the school you would like to go to. Make a quick drawing of some of these students. What are the students doing? Tell me three things about these students.  The adults. Think about some of the adults at the school you would like to go to. Make a quick drawing of some of these adults. What are the adults doing? Tell me three things about these adults.  Me. Think about the kind of school you would like to go to. Make a quick drawing of what you would be doing at this school. Tell me three things about the way you feel at this school. |
| --- |

**Supplementary Table 2.** Parent semi-structured interview schedule (including primary questions and prompt questions).

| 1. Tell me about your child.   1. How would you describe him/her? 2. What are his/her strengths?   2. Current school: Tell me about your child’s current school.   1. How long has your son/daughter been coming to this school? 2. How does your child feel about coming to this school? Did they have a choice about coming to this school? 3. In what ways does this school support your son/daughter? 4. Is there anything else you think the school could do to support your son/daughter? 5. Since coming to this school, has anything else in their life outside of school changed?   3. Previous school: Tell me about your child’s previous school(s).   1. How long was your son/daughter at their previous school? 2. What was your son/daughter’s experience like in their previous school? 3. How long was your son/daughter at their previous school? 4. What was your son/daughter’s experience like in their previous school? 5. What worked well in your child’s previous school? What support did they receive? Was it helpful? 6. What didn’t work so well in your child’s previous school?   4. Exclusion: Tell me about what happened when your son/daughter moved from (previous school) to (current school)   1. What led to your child being moved/excluded from school? 2. What did the experience feel like for you? And what did it seem like for your son/daughter? 3. What people were involved in your child’s move/exclusion 4. Who supported you during your child’s move/exclusion?    1. What did they do to support you? 5. How did your son/daughter’s move/exclusion affect your family? 6. Do you feel the move to a new provision was successful for your son/daughter? Why/why not?    1. What made it successful?    2. What could have helped to make the move better for you? 7. How long did it take for your child to move schools/find a new school?    1. What impact do you think this had on you and your child?   5. Hope and Aspirations: Tell me about your hopes for your son/daughter future?   1. How long will your son/daughter be in this school? 2. What are your aspirations for your son/daughter?    1. What might help them get there?    2. What kind of support, if any, will they need to achieve their goals in life?    3. What are your son/daughter’s aspirations?   That’s all my questions. Are there any issues that we haven’t covered that you think are important? |
| --- |

**Supplementary Table 3.** Teacher semi-structured interview schedule

(including primary questions and prompt questions).

| 1. Current school: To begin, could you tell me about your role in the school.   - - 1. How long have you been working with pupil x? What are his/her strengths? What do you think s/he finds difficult or particularly challenging? Where do you think s/he needs the most support? What was your experience working with pupil x when he/she first started at the school? (if relevant) What is your experience working with pupil x now?     2. What do you think pupil x likes about this school?     3. What do you think pupil x finds difficult in school?     4. How do you support x? Do you think this support can be maintained over time?     5. How does your school support pupil x?     6. Is there anything else you think the school could do to support pupil x?     7. In what ways do you work with x’s parents? How often do you make contact with x’s parents? How involved are x’s parents in his/her education?     8. Is this provision seen as a temporary measure or an option for the reminder of their school career?  1. How is the decision made? 2. Was that always the plan? 3. Is there a plan for what happens next? 4. Where do you see X in the future?   Autism: To finish up, I just have some overall questions about autism.   1. How would you describe autism? 2. Are there many autistic pupils in your school? 3. How much experience have you had supporting pupils on the autism spectrum? Do you feel well-equipped to do so? How could you be better supported? 4. What do you think your school does to support the needs of autistic pupils? Should it do less? More?   That’s all my questions. Are there any issues that we haven’t covered that you think are important? |
| --- |

**Supplementary Table 4.** Educational psychologist semi-structured interview schedule .

| Current role: To begin, could you tell me about your role in schools?   1. In what ways do you support the inclusion of autistic pupils in mainstream schools? 2. Is there any other ways you think Educational Psychologists can support the inclusion of autistic pupils in mainstream schools? 3. What do you think the county does to support the inclusion of autistic pupils in mainstream schools? 4. Is there anything else you think the county could do to support these pupils? 5. Do you think there are any barriers for autistic pupils accessing mainstream schools? 6. Have you worked with many autistic pupils who have been excluded or managed moved from school? 7. What have been the main reasons for the exclusion or managed move of these pupils? 8. What do you think the exclusion experience has been like for autistic young people? 9. How have you supported autistic pupils who have been excluded or managed moved? 10. How do you support autistic pupils who are at risk of exclusion? 11. Are there any other ways you think Educational Psychologists could support these pupils through the exclusion or managed move?   That’s all my questions. Are there any issues that we haven’t covered that you think are important? |
| --- |

**Supplementary Table 5.** Specialist autism teacher semi-structured interview schedule.

| Current role:   - - 1. To begin could you tell me about your role in schools?     2. How much contact do you have with young people?     3. How much contact do you have with parents     4. Can you tell me what the term ‘inclusion’ means to you?     5. In what ways do you support the inclusion of autistic pupils in mainstream schools? What do you think works/doesn’t work?     6. What are the barriers, if any, for autistic pupils accessing mainstream schools?     7. Are there any other ways you think the specialist teachers should support the inclusion of autistic pupils in mainstream schools?     8. What do you think the county does to support the inclusion of autistic pupils in mainstream schools? What works/doesn’t work?     9. Is there anything else you think the county should do to support these pupils?     10. Have you worked with many autistic pupils who have been excluded or managed moved from school? Can you give me an example of a case (without identifying the children/parents/schools involved?     11. What have been the main reasons for the exclusion or managed move of these pupils? At what point did you (as a specialist teacher) get involved? What could have been done in these cases to support children’s inclusion?     12. What do you think is the impact of being excluded for autistic young people?     13. How have you supported autistic pupils who have been excluded or managed moved?     14. How do you support autistic pupils who are at risk of exclusion? What do you think has worked/has not worked?     15. Are there any other ways you think specialist teachers could support these pupils through the exclusion or managed move?   That’s all my questions. Are there any issues that we haven’t covered that you think are important? |
| --- |
